# Symptom screening rules to identify active pulmonary tuberculosis: Findings from the Zambian South African Tuberculosis and HIV/AIDS Reduction (ZAMSTAR) trial prevalence surveys

**DOI:** 10.1371/journal.pone.0172881

**Published:** 2017-03-03

**Authors:** M. M. Claassens, C. van Schalkwyk, S. Floyd, H. Ayles, N. Beyers

**Affiliations:** 1 Desmond Tutu TB Centre, Department of Pediatrics and Child Health, Stellenbosch University, Cape Town, South Africa; 2 The South African Department of Science and Technology/National Research Foundation Centre of Excellence in Epidemiological Modelling and Analysis, Stellenbosch University, Stellenbosch, South Africa; 3 Department of Clinical Research, London School of Hygiene and Tropical Medicine, London, United Kingdom; 4 Zambia AIDS Related Tuberculosis Project, University of Zambia Ridgeway Campus, Lusaka, Zambia; McGill University, CANADA

## Abstract

**Background:**

High tuberculosis (TB) burden countries should consider systematic screening among adults in the general population. We identified symptom screening rules to be used in *addition* to cough ≥2 weeks, in a context where X-ray screening is not feasible, aiming to increase the sensitivity of screening while achieving a specificity of ≥85%.

**Methods:**

We used 2010 Zambia South Africa Tuberculosis and HIV/AIDS Reduction (ZAMSTAR) survey data: a South African (SA) training dataset, a SA testing dataset for internal validation and a Zambian dataset for external validation. Regression analyses investigated relationships between symptoms or combinations of symptoms and active disease. Sensitivity and specificity were calculated for candidate rules.

**Results:**

Among all participants, the sensitivity of using only cough ≥2 weeks as a screening rule was less than 25% in both SA and Zambia. The addition of any three of six TB symptoms (cough <2 weeks, night sweats, weight loss, fever, chest pain, shortness of breath), or 2 or more of cough <2 weeks, night sweats, and weight loss, increased the sensitivity to ~38%, while reducing specificity from ~95% to ~85% in SA and ~97% to ~92% in Zambia. Among HIV-negative adults, findings were similar in SA, whereas in Zambia the increase in sensitivity was relatively small (15% to 22%).

**Conclusion:**

High TB burden countries should investigate cost-effective strategies for systematic screening: one such strategy could be to use our rule in addition to cough ≥2 weeks.

## Introduction

A person presumed to have pulmonary tuberculosis (TB) is currently defined as someone with an unexplained cough for ≥2 weeks or with unexplained findings on chest radiograph suggestive of TB [1], irrespective of their HIV status or any other individual characteristic. Systematic screening for active TB in the community tends to find more cases earlier in the disease progression compared to when symptomatic patients seek healthcare [2]. A systematic review [3] developed a standardized screening rule for excluding TB in individuals who are human immunodeficiency virus (HIV) positive in resource poor environments with the primary aim of achieving a high negative predictive value, which would allow initiation of isoniazid preventive therapy (IPT) to few “false negative” TB cases. The rule indicated an HIV-positive individual with any of current cough, night sweats, weight loss or fever should not be offered IPT before further investigation for TB. This rule is now part of World Health Organization (WHO) guidelines [4].

To date, few TB prevalence surveys from high HIV burden settings in sub-Saharan Africa have been used to evaluate symptom screening rules. A western Kenyan survey showed a sensitivity of 41% and 82% respectively for cough ≥2 weeks and any TB symptom (cough, haemoptysis, fever, night sweats, weight loss, of any duration or severity) amongst HIV-negative individuals, and 69% and 96% respectively among HIV-positive individuals [5]. Specificity could not be stratified by HIV status (HIV status of TB negative individuals was not collected) and was 89% and 32% respectively for cough ≥2 weeks and any TB symptom. However, in this survey, sputum cultures were only conducted if a participant was screened positively by symptom, smear or chest x-ray. The Zambia South Africa Tuberculosis and HIV/AIDS Reduction (ZAMSTAR) prevalence surveys [6–8] are unusual: all participants provided a sputum sample for culture, irrespective of TB symptoms and without X-ray screening. These surveys provide an opportunity to investigate the performance of alternative symptom screening rules in a high TB/HIV burden setting although it is expected that sensitivity would be lower than for the Kenyan survey for instance, because all participants had sputum cultures and not only those with a positive screen.

The impact of systematic screening on the epidemiology of TB depends on the frequency of screening and the sensitivity of the screening method [9]. For instance, when screening annually using a method with 50% sensitivity, the transmission rate could be decreased by up to 27%, depending on the cure rate and the case detection rate. Currently there is emphasis on systematic screening in programmatic settings in order to identify more TB cases earlier [10]. Since cough ≥2 weeks is used as the screening symptom, for our analysis we focused on the group not eligible for screening or diagnostic algorithms. We used ZAMSTAR data to develop symptom screening rules for the general population regardless of HIV status and for the HIV-negative population with the aim to increase screening but not diagnostic accuracy. We describe how the rules to be used in *addition* to cough ≥2 weeks were developed and validated, to increase the sensitivity of systematic screening in a high TB/HIV burden setting while at the same time achieving a specificity of at least 85%. We chose 85% on the basis that it would not be feasible to implement screening if >15% of the population screened positive, and were thus eligible for further investigation for TB, among those without TB, i.e. to ensure some limit on the programmatic resources that would be required to manage “false-positive” cases. The choice was also made in the context that in our setting the currently recommended screening rule of cough > = 2 weeks had a specificity of ~95%, so our choice corresponded to an aim to identify a rule for which specificity was < = 10% lower than the currently recommended screen.

## Methods

### Case definition

A case of culture positive prevalent TB was defined as a participant who had a positive culture for *Mycobacterium tuberculosis (M*.*tb)*. One sputum sample collected from each participant was split and cultured in two Mycobacteria Growth Indicator Tubes (Becton, Dickinson and Company, Franklin Lakes, New Jersey, US) [6]. Positive cultures were speciated according to the study algorithm as *M*.*tb*. A speciated non-tuberculous mycobacterium was defined not to be TB. Participants whose sample was lost, or for whom both cultures were contaminated, were excluded.

### Setting and study population

Twenty-four communities were surveyed (eight in SA and 16 in Zambia) in a TB/HIV prevalence survey conducted in 2010 to measure the primary endpoint of the ZAMSTAR trial [6–8]. All individuals aged ≥18 years who stayed in households in the previous 24 hours were asked to participate. All participants had a respiratory secretion sample collected. 90,601 participants were enrolled and 894 cases were diagnosed out of 64,463 participants whose sputum sample was evaluable for *M*.*tb*, 702/30,017 in SA and 192/34,446 in Zambia. In Zambia, the average of the 16 community-specific prevalences was 555/100,000 among participants with an evaluable sample (range 221 to 1,095/100,000). In SA, the average of the eight community-specific prevalences was 2,338/100,000 among participants with an evaluable sample (range 1,489 to 3,103/100,000).

### Data collection

A structured questionnaire was used by trained, supervised research assistants to collect socio-demographic information and to elicit symptoms from participants. Each participant was asked if they had a current cough, and if yes they were asked for how many weeks they had been coughing. It was therefore possible to distinguish participants who had a cough of ≥2 weeks. Other symptoms included currently producing phlegm/sputum or blood, current shortness of breath, sweating at night or fever and weight loss within the past month.

A respiratory secretion sample was collected on the spot spontaneously or with the assistance of breathing techniques. The laboratory algorithm has been described elsewhere [6]. HIV testing was done on all participants who consented, using rapid HIV test kits and a finger-prick sample. Participants were also asked to self-report their HIV status if known. If participants did not give consent to be tested for HIV, their self-reported HIV status was used in the analysis.

### Data analysis

The screening rules were developed and internally validated for symptomatic participants in *addition* to cough ≥2 weeks by randomly dividing the SA dataset into equal sized training and testing datasets, and then externally validated with the Zambian data to comply with the “presumed TB case” definition recommended by WHO. Six symptoms, namely cough <2 weeks, night sweats, fever, chest pain, weight loss and shortness of breath, were investigated for association with prevalent TB among participants *without* a cough ≥2 weeks. Logistic regression was used to identify which of these symptoms, or combinations of these symptoms, were associated with prevalent TB in the South African dataset (results shown in supporting information). The association of different counts of symptoms, still excluding those with cough ≥2 weeks, were similarly investigated: first among all six TB symptoms, second restricted to the four symptoms considered in the 2013 WHO guidelines [4] (cough, night sweats, weight loss and fever), and third restricted to three of these four symptoms which were most strongly associated with prevalent TB in this dataset–cough, night sweats and weight loss. From these analyses, a few alternative candidate screening rules were identified, i.e. that met our pre-specified criteria of having a specificity of at least 85%.

Using only the training dataset, the overall values of sensitivity and specificity for each candidate screening rule used in combination with cough ≥2 weeks were calculated. We calculated 95% confidence intervals based on bootstrapping of the data, stratified on community and with clustering by census enumeration area, to account for the sampling design. We repeated the calculation of sensitivities and specificities with the SA testing dataset for internal validation and with the Zambian dataset for external validation of the alternative rules. We then repeated the calculation of sensitivities and specificities of the screening rules, but with restriction to HIV-negative participants. We also repeated the analyses with restriction to HIV-positive participants for completeness—these analyses are not included because WHO guidelines for TB screening among HIV-positive individuals are established based on a meta-analysis of various studies including the data from the HIV-positive participants in the 2005 ZAMSTAR Zambian survey [7] which made up 27% of the weight of the meta-analysis [3].

### Ethics

Stellenbosch University, the University of Zambia and the London School of Hygiene and Tropical Medicine gave ethics approval. Written informed consent was obtained from participants.

## Results

Among all 90,601 participants, age and gender were recorded for 98.7% (57,075/57,089) in Zambia and 99.9% (32,770/32,792) in SA; among these the percentages who gave a blood sample for HIV testing were 67.1% (38,300/57,075) and 34.0% (11,147/32,770) respectively. For each community, HIV prevalence among participants who gave a blood sample for HIV testing was age-sex standardized to the overall participant population. In Zambia, the average of the 16 community-specific prevalences was 17.1% (range 8.1% to 26.6%). In SA, the average of the eight community-specific prevalences was 18.3% (range 14.2% to 22.9%).

Among participants with a sputum sample that was evaluable for *M*.*tb* (n = 64,463), 10,165/30,017 from SA and 23,480/34,446 from Zambia gave a blood sample for HIV testing. There were no missing data on reported symptoms, since electronic data capture forced a yes or no answer. There were missing data on duration of cough (3.1%) and these participants were removed from analyses.

18,414/30,017 participants from SA and 28,990/34,446 participants from Zambia either gave a blood sample for HIV testing or reported they had previously tested for HIV and reported their HIV status based on their most recent HIV test; the percentages who were HIV-positive were 16.1% (2,965/18,414) in SA and 17.2% (4,974/28,990) in Zambia. In SA, 144/1,670 (8.6%) with a cough ≥2 weeks had TB compared with 555/28,289 (2.0%) without a cough ≥2 weeks (OR 4.7, 95%CI 4.0–5.5) while in Zambia, 46/1,175 (3.9%) with a cough ≥2 weeks had TB compared with 143/33,132 (0.4%) without a cough ≥2 weeks (OR 9.4, 95% confidence interval 6.6–13.4).

In Table 1 the SA and Zambian surveys are compared, showing the demographics of participants regardless of HIV status and of participants known to be HIV negative. In the entire SA sample for those participants regardless of HIV status, the TB prevalence was highest among participants who currently coughed (6.0%), followed by recent unintentional weight loss (5.0%) and current shortness of breath (4.8%). In Zambia, the TB prevalence was highest among participants who had current fever (2.5%), followed by current night sweats (2.1%) and current cough (2.1%). In SA, the highest TB prevalence among participants known to be HIV negative was for those who currently coughed (5.3%), followed by recent unintentional weight loss (4.3%) and current night sweats (4.2%). In Zambia, the highest prevalence was among those who had current shortness of breath (1.2%), followed by current chest pains (1.1%) and current cough (1.0%).

**Table 1:** Demographics of ZAMSTAR prevalence survey participants in South Africa and Zambia, 2010.

		**South Africa**			**Zambia**		
**All participants, regardless of HIV status**							
		**Total**	**TB disease**	**%**	**Total**	**TB disease**	**%**
**Total**		30017	702	2.3%	34446	192	0.6%
**Sex**	Male	11297	333	2.9%	11638	92	0.8%
	Female	18720	369	2.0%	22808	100	0.4%
**Age**	15–24	8819	169	1.9%	12169	45	0.4%
	25–34	9167	197	2.1%	10129	82	0.8%
	35–44	5577	141	2.5%	5196	42	0.8%
	45–54	3480	107	3.1%	3154	12	0.4%
	55+	2974	88	3.0%	3798	11	0.3%
**Current cough**	No	26152	469	1.8%	30444	106	0.3%
	Yes	3865	233	6.0%	4002	86	2.1%
**Duration of cough**	<2 weeks	2137	86	4.0%	2688	37	1.4%
	> = 2 weeks	1670	144	8.6%	1175	46	3.9%
	Unknown duration	58	3	5.2%	139	3	2.2%
**Current fever**	No	24451	522	2.1%	32578	145	0.4%
	Yes	5566	180	3.2%	1868	47	2.5%
**Current shortness of breath**	No	27444	578	2.1%	32375	151	0.5%
	Yes	2573	124	4.8%	2071	41	2.0%
**Current night sweats**	No	25159	484	1.9%	32431	150	0.5%
	Yes	4858	218	4.5%	2015	42	2.1%
**Recent unintentional weight loss**	No	25658	486	1.9%	29979	129	0.4%
	Yes	4359	216	5.0%	4467	63	1.4%
**Current chest pains**	No	26865	559	2.1%	31568	140	0.4%
	Yes	3152	143	4.5%	2878	52	1.8%
**Participants known to be HIV negative**							
		**Total**	**TB disease**	**%**	**Total**	**TB disease**	**%**
**Total**		15449	302	2.0%	24016	86	0.4%
**Sex**	Male	5344	144	2.7%	8079	41	0.5%
	Female	10105	158	1.6%	15937	45	0.3%
**Age**	15–24	4738	89	1.9%	9615	30	0.3%
	25–34	4470	64	1.4%	6905	34	0.5%
	35–44	2749	52	1.9%	2984	12	0.4%
	45–54	1809	51	2.8%	1975	3	0.2%
	55+	1683	46	2.7%	2537	7	0.3%
**Current cough**	No	13382	193	1.4%	21490	60	0.3%
	Yes	2067	109	5.3%	2526	26	1.0%
**Duration of cough**	<2 weeks	1157	35	3.0%	1774	12	0.7%
	> = 2 weeks	873	72	8.2%	666	13	2.0%
	Unknown duration	37	2	5.4%	86	1	1.2%
**Current fever**	No	12413	218	1.8%	22865	76	0.3%
	Yes	3036	84	2.8%	1151	10	0.9%
**Current shortness of breath**	No	14028	245	1.7%	22730	71	0.3%
	Yes	1421	57	4.0%	1286	15	1.2%
**Current night sweats**	No	12902	196	1.5%	22816	77	0.3%
	Yes	2547	106	4.2%	1200	9	0.8%
**Recent unintentional weight loss**	No	13117	202	1.5%	21186	71	0.3%
	Yes	2332	100	4.3%	2830	15	0.5%
**Current chest pains**	No	13753	235	1.7%	22142	66	0.3%
	Yes	1696	67	4.0%	1874	20	1.1%

HIV: human immunodeficiency virus; TB: tuberculosis

When considering a count of symptoms (Table 2), in the entire SA sample, the TB prevalence was highest among participants regardless of HIV status who had 3 of cough <2 weeks, night sweats or weight loss (CSW) (7.5%), followed by 4 of cough <2 weeks, night sweats, fever or weight loss (CSFW) (6.9%). In Zambia, the TB prevalence was highest among participants who had 4 of CSFW (3.9%), followed by 3 out of 6 symptoms (2.5%). In SA, the highest TB prevalence among participants known to be HIV negative was for those who had 3 of CSW (5.1%), followed by 4 of CSFW (4.3%). In Zambia, the highest prevalence was among those who had 4 of CSFW (3.4%), followed by 3 of CSW (1.5%).

**Table 2:** Counts of symptoms in ZAMSTAR prevalence survey participants who did not have a cough ≥2 weeks, in South Africa and Zambia, 2010.

		South Africa				Zambia			
**All participants, regardless of HIV status**									
** **		**% with symptom**	**Total**	**TB**	**%**	**% with symptom**	**Total**	**TB**	**%**
**Total**			28289	555	2.0%		33132	143	0.4%
**Count of symptoms**	0	64.3%	18190	291	1.6%	72.3%	23952	74	0.3%
	1	17.7%	5013	108	2.2%	18.3%	6049	25	0.4%
	2	9.7%	2741	63	2.3%	6.1%	2025	21	1.0%
	3	4.8%	1352	39	2.9%	2.2%	727	18	2.5%
	4+	3.5%	993	54	5.4%	1.1%	379	5	1.3%
**Count of symptoms, among cough <2 weeks, weight loss, night sweats (CSW)**	0	74.1%	20959	336	1.6%	78.7%	26058	81	0.3%
	1	18.3%	5169	119	2.3%	17.8%	5894	41	0.7%
	2	6.6%	1869	78	4.2%	3.2%	1068	19	1.8%
	3	1.0%	292	22	7.5%	0.3%	112	2	1.8%
**Count of 4 symptoms, i.e. cough <2weeks, weight loss, night sweats, fever (CWSF)**	0	67.4%	19067	305	1.6%	76.8%	25460	79	0.3%
	1	18.8%	5313	118	2.2%	17.8%	5887	32	0.5%
	2	9.6%	2708	71	2.6%	4.2%	1384	24	1.7%
	3	3.5%	997	47	4.7%	1.1%	350	6	1.7%
	4	0.7%	204	14	6.9%	0.2%	51	2	3.9%
**Participants known to be HIV negative**									
** **		**% with symptom**	**Total**	**TB**	**%**	**% with symptom**	**Total**	**TB**	**%**
**Total**			14539	228	1.6%		23264	72	0.3%
**Count of symptoms**	0	61.9%	8999	107	1.2%	73.4%	17069	50	0.3%
	1	19.2%	2788	50	1.8%	18.3%	4252	13	0.3%
	2	10.4%	1515	31	2.0%	5.6%	1304	3	0.2%
	3	5.0%	723	22	3.0%	1.8%	427	5	1.2%
	4+	3.5%	514	18	3.5%	0.9%	212	1	0.5%
**Count of symptoms, among cough <2 weeks, weight loss, night sweats (CSW)**	0	72.7%	10570	127	1.2%	79.9%	18593	55	0.3%
	1	19.7%	2864	57	2.0%	17.1%	3987	15	0.4%
	2	6.7%	969	37	3.8%	2.7%	619	1	0.2%
	3	0.9%	136	7	5.1%	0.3%	65	1	1.5%
**Count of 4 symptoms, i.e. cough <2 weeks, weight loss, night sweats, fever (CWSF)**	0	65.3%	9499	112	1.2%	78.1%	18162	54	0.3%
	1	20.2%	2934	60	2.0%	17.4%	4048	15	0.4%
	2	10.4%	1516	34	2.2%	3.6%	836	2	0.2%
	3	3.4%	498	18	3.6%	0.8%	189	0	0.0%
	4	0.6%	92	4	4.3%	1.4%	29	1	3.4%

HIV: human immunodeficiency virus; TB: tuberculosis

**Fig 1:**
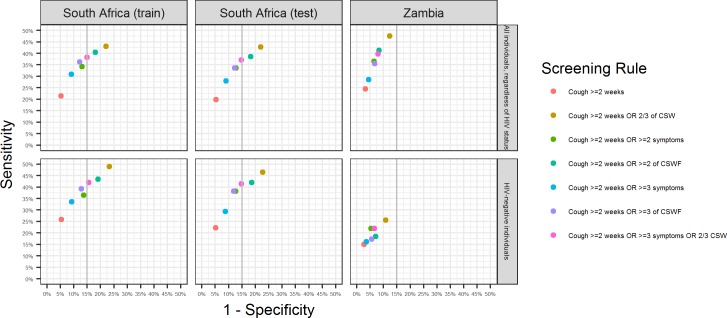
Sensitivity and specificity for selected screening rules in all individuals regardless of HIV status and in HIV negative individuals, in South Africa and Zambia. Symptoms: cough <2 weeks, night sweats, weight loss, fever, chest pain, shortness of breath; CSW: cough <2 weeks, night sweats, weight loss; CSWF: cough <2 weeks, night sweats, weight loss, fever.

Table 3 shows the sensitivity and specificity for all screening rules that were considered using the training dataset, separately for each of the training, testing, and validation datasets. Fig 1 shows sensitivity and specificity for 3 screening rules that met pre-specified criteria (specificity ≥85%) in the SA training dataset, as well as for a few alternative screening rules that were relatively close to meeting pre-specified criteria. Including all participants regardless of HIV status, using cough ≥2 weeks to identify those who should be further investigated for TB had a sensitivity of 21% (95%CI 17–26%) in the SA training dataset, a sensitivity of 20% (95%CI 16–24%) in the SA testing dataset and 24% (95%CI 18–30%) in Zambia, with specificity of 95% in SA (training and testing), and 97% in Zambia. Adding three or more of six TB symptoms or 2 or more of cough <2 weeks, night sweats or weight loss (CSW) to this screen increased sensitivity to 38% (95%CI 33–43%) in SA (training), 37% (95%CI 32–42%) in SA (testing) and 40% (95%CI 32–46%) in Zambia, with specificity falling to 85% (SA training and testing) and 92% (Zambia).

**Table 3:** Screening rules’ sensitivity and specificity in South Africa (training and testing datasets) and Zambia 2010 prevalence surveys.

	South Africa—train						South Africa—test						Zambia					
	Sensitivity	2.5%	97.5%	Specificity	2.5%	97.5%	Sensitivity	2.5%	97.5%	Specificity	2.5%	97.5%	Sensitivity	2.5%	97.5%	Specificity	2.5%	97.5%
**All individuals, regardless of HIV status**																		
Cough ≥2 weeks*	**21.4**	**17.2**	**25.6**	**94.8**	**94.3**	**95.0**	**19.8**	**15.7**	**24.0**	**94.7**	**94.2**	**94.9**	**24.4**	**18.1**	**30.1**	**96.7**	**96.1**	**96.7**
*Cough ≥2 weeks OR ≥1 symptoms* *	*57*.*8*	*52*.*6*	*62*.*9*	*61*.*1*	*60*.*3*	*61*.*8*	*58*.*9*	*53*.*4*	*63*.*7*	*61*.*2*	*60*.*3*	*61*.*8*	*60*.*9*	*53*.*0*	*66*.*7*	*70*.*0*	*69*.*2*	*70*.*2*
*Cough ≥2 weeks OR ≥2 symptoms*	*43*.*0*	*37*.*8*	*48*.*2*	*77*.*9*	*77*.*1*	*78*.*4*	*42*.*8*	*37*.*4*	*47*.*7*	*78*.*0*	*77*.*1*	*78*.*4*	*47*.*6*	*40*.*0*	*53*.*8*	*87*.*6*	*86*.*9*	*87*.*6*
**Cough ≥2 weeks OR *≥*3 symptoms**	**34.2**	**29.4**	**39.1**	**86.9**	**86.3**	**87.3**	**33.6**	**28.5**	**38.5**	**87.2**	**86.5**	**87.6**	**36.5**	**29.3**	**42.8**	**93.5**	**92.9**	**93.5**
*Cough ≥2 weeks OR ≥1 of CSWF*	*56*.*1*	*50*.*9*	*61*.*1*	*64*.*2*	*63*.*3*	*64*.*8*	*56*.*6*	*51*.*1*	*61*.*4*	*64*.*1*	*63*.*2*	*64*.*6*	*58*.*2*	*50*.*3*	*64*.*2*	*74*.*4*	*73*.*7*	*74*.*5*
*Cough ≥2 weeks OR ≥2 of CSWF*	*40*.*5*	*35*.*3*	*45*.*6*	*81*.*9*	*81*.*2*	*82*.*4*	*38*.*5*	*33*.*2*	*43*.*4*	*81*.*8*	*81*.*0*	*82*.*2*	*41*.*3*	*33*.*7*	*47*.*5*	*91*.*6*	*90*.*9*	*91*.*6*
**Cough ≥2 weeks OR *≥*3 of CSWF**	**30.8**	**26.1**	**35.5**	**90.9**	**90.2**	**91.2**	**27.9**	**23.1**	**32.4**	**90.9**	**90.3**	**91.2**	**28.6**	**21.8**	**34.6**	**95.5**	**94.9**	**95.5**
*Cough ≥2 weeks OR ≥1 of CSW*	*51*.*6*	*46*.*3*	*56*.*7*	*70*.*4*	*69*.*6*	*71*.*0*	*52*.*3*	*46*.*9*	*57*.*2*	*70*.*6*	*69*.*7*	*71*.*1*	*56*.*2*	*49*.*3*	*63*.*2*	*75*.*8*	*75*.*4*	*76*.*3*
**Cough ≥2 weeks OR 2 or 3 of CSW**	**36.2**	**31.3**	**41.1**	**87.8**	**87.1**	**88.1**	**33.6**	**28.6**	**38.4**	**87.7**	**87.0**	**88.1**	**35.5**	**28.4**	**41.6**	**93.3**	**92.7**	**93.3**
**Cough ≥2 weeks OR all of CSW**	**25.9**	**21.5**	**30.4**	**93.9**	**93.3**	**94.1**	**21.6**	**17.3**	**25.8**	**93.8**	**93.2**	**94.0**	**25.4**	**19.0**	**31.1**	**96.4**	**95.8**	**96.4**
*Cough ≥2 weeks OR ≥3 symptoms OR ≥2 of CSWF*	*40*.*7*	*35*.*7*	*45*.*8*	*81*.*3*	*80*.*5*	*81*.*7*	*39*.*7*	*34*.*3*	*44*.*6*	*81*.*3*	*80*.*6*	*81*.*8*	*43*.*9*	*36*.*4*	*50*.*3*	*91*.*1*	*90*.*4*	*91*.*1*
**Cough ≥2 weeks OR *≥*3 symptoms OR 2/3 CSW**	38.2	33.2	43.2	85.0	84.3	85.4	37.1	31.9	42.0	85.2	84.5	85.6	39.7	32.3	46.0	92.0	91.4	92.0
**Individuals known to be HIV negative**																		
Cough ≥2 weeks*	**25.9**	**19.0**	**33.1**	**94.6**	**93.9**	**94.9**	**22.3**	**16.0**	**28.7**	**94.8**	**94.0**	**95.1**	**15.0**	**7.8**	**23.1**	**96.9**	**96.7**	**97.1**
*Cough ≥2 weeks OR ≥1 symptoms* *	*62*.*9*	*54*.*4*	*70*.*3*	*59*.*0*	*57*.*9*	*60*.*0*	*65*.*6*	*57*.*7*	*72*.*4*	*58*.*6*	*57*.*5*	*59*.*5*	*40*.*7*	*30*.*2*	*51*.*2*	*71*.*1*	*70*.*6*	*71*.*7*
*Cough ≥2 weeks OR ≥2 symptoms*	*49*.*0*	*40*.*7*	*56*.*7*	*76*.*7*	*75*.*6*	*77*.*4*	*46*.*5*	*38*.*4*	*53*.*9*	*77*.*3*	*76*.*2*	*77*.*9*	*25*.*5*	*16*.*5*	*34*.*9*	*88*.*8*	*88*.*4*	*89*.*2*
**Cough ≥2 weeks OR *≥*3 symptoms**	**36.4**	**28.6**	**44.0**	**86.3**	**85.4**	**86.9**	**38.2**	**30.4**	**45.5**	**87.3**	**86.3**	**87.8**	**22.0**	**13.6**	**31.2**	**94.3**	**94.0**	**94.6**
*Cough ≥2 weeks OR ≥1 of CSWF*	*61*.*5*	*53*.*1*	*69*.*0*	*62*.*2*	*61*.*1*	*63*.*2*	*63*.*7*	*55*.*8*	*70*.*6*	*62*.*0*	*60*.*8*	*62*.*9*	*36*.*0*	*25*.*9*	*46*.*4*	*75*.*7*	*75*.*1*	*76*.*2*
*Cough ≥2 weeks OR ≥2 of CSWF*	*43*.*4*	*35*.*2*	*51*.*2*	*80*.*9*	*79*.*8*	*81*.*5*	*42*.*0*	*34*.*1*	*49*.*4*	*81*.*4*	*80*.*4*	*82*.*1*	*18*.*5*	*10*.*6*	*27*.*3*	*92*.*5*	*92*.*2*	*92*.*8*
**Cough ≥2 weeks OR *≥*3 of CSWF**	**33.6**	**26.0**	**41.1**	**90.7**	**89.8**	**91.1**	**29.3**	**22.1**	**36.2**	**91.2**	**90.3**	**91.6**	**16.1**	**8.8**	**24.4**	**96.0**	**95.8**	**96.3**
*Cough ≥2 weeks OR ≥1 of CSW*	*56*.*6*	*48*.*1*	*64*.*3*	*69*.*3*	*68*.*2*	*70*.*2*	*58*.*6*	*50*.*4*	*65*.*7*	*68*.*9*	*67*.*7*	*69*.*7*	*34*.*9*	*25*.*0*	*45*.*0*	*77*.*5*	*77*.*0*	*78*.*0*
**Cough ≥2 weeks OR 2 or 3 of CSW**	**39.2**	**31.2**	**47.0**	**87.2**	**86.3**	**87.8**	**38.2**	**30.4**	**45.6**	**88.1**	**87.2**	**88.6**	**17.3**	**9.7**	**25.8**	**94.1**	**93.8**	**94.4**
**Cough ≥2 weeks OR all of CSW**	**28.0**	**20.8**	**35.4**	**93.7**	**93.0**	**94.0**	**24.8**	**18.1**	**31.4**	**94.0**	**93.2**	**94.3**	**16.1**	**8.8**	**24.4**	**96.6**	**96.4**	**96.9**
*Cough ≥2 weeks OR ≥3 symptoms OR ≥2 of CSWF*	*44*.*8*	*36*.*6*	*52*.*6*	*80*.*2*	*79*.*1*	*80*.*9*	*43*.*3*	*35*.*3*	*50*.*7*	*80*.*8*	*79*.*7*	*81*.*4*	*23*.*2*	*14*.*5*	*32*.*5*	*92*.*1*	*91*.*8*	*92*.*5*
Cough ≥2 weeks OR *≥*3 symptoms OR 2/3 CSW	*42*.*0*	*33*.*8*	*49*.*7*	*84*.*4*	*83*.*4*	*85*.*0*	**41.4**	**33.5**	**48.8**	**85.2**	**84.2**	**85.7**	**22.0**	**13.6**	**31.2**	**93.0**	**92.7**	**93.3**

Symptoms: cough <2 weeks, night sweats, weight loss, fever, chest pain, shortness of breath; CSW: cough <2 weeks, night sweats, weight loss; CSWF: cough <2 weeks, night sweats, weight loss, fever; HIV: human immunodeficiency virus; TB: tuberculosis; CI: confidence interval

* Rules shown in bold met the specificity criteria of ≥85% and rules shown in italics were added for completeness.

In participants known to be HIV negative using cough ≥2 weeks to identify those who should be further investigated for TB had a sensitivity of 26% (95%CI 19–33%) in the SA training dataset, a sensitivity of 22% (95%CI 16–29%) in the SA testing dataset and 15% (95%CI 8–23%) in Zambia, with specificity of about 95% in SA (training and testing) and 97% in Zambia. Adding three or more of six TB symptoms or 2 or more of CSW to this screen increased sensitivity to 42% (95%CI 34–50%) in SA (training), 41% (95%CI 34–49%) in SA (testing) and 22% (95%CI 14–31%) in Zambia, with specificity falling to 84% (SA training), 85% (SA testing) and 93% (Zambia).

## Discussion

WHO systematic screening algorithms for active TB include an interview about HIV status especially in settings with a high HIV prevalence [11]. Our analysis, for HIV-negative individuals, contributes information about symptom screening rules in addition to cough ≥2 weeks in a setting where TB prevalence is high in the general population and not only among risk groups.

Few studies have used TB prevalence survey data to develop symptom screening rules for use in the general population—there is a scarcity of data from settings with both high TB and HIV prevalence. A review on HIV-negative individuals and individuals with unknown HIV status [12] did not look at individual participant data meta-analysis or the development of a standardized screening rule, but calculated summary estimates of sensitivity and specificity for consistent screening definitions across studies: cough of any duration, prolonged cough (≥2 or ≥3 weeks, depending on the study), and any TB symptom out of ≥3 questions asked. Our findings were similar to a study in SA miners [13] which showed a sensitivity of around 30% for 2 out of 3 among CSW but contrasted with findings from Zimbabwe [14], where the symptom screening rule performed better with regards to sensitivity and specificity than any of the rules from the ZAMSTAR data and the ZAMSTAR rules had worse specificity for a screen of any TB symptom.

Our study confirmed that symptom screening alone will miss a large proportion of individuals with active prevalent TB in the general population. However, a screening rule of three of six symptoms, or two of CSW, in addition to cough ≥2 weeks increased the sensitivity of symptom screening in the general population while maintaining a specificity of ≥84%, in SA and Zambia. The rationale for maintaining a specificity of ≥84% is simple: a high specificity limits the number of individuals whose screening result is false positive and who will be investigated further for TB with sputum microscopy, culture and/or Xpert MTB/RIF (Cepheid, Sunnyvale, CA, USA). Increasing the sensitivity of symptom screening should mean that fewer true cases are missed, ensuring more TB cases are referred for TB treatment, potentially decreasing transmission within communities [15]. Our screening rule would improve sensitivity and specificity beyond what is currently used, for example in the HPTN 071 (PopART, Population effects of Antiretroviral Therapy to reduce HIV transmission) study [16].

Survey participants from an area of Cape Town with a low HIV prevalence [17] were not tested for HIV, but all had sputum smears, cultures and chest x-rays. The survey showed a low sensitivity for using any one symptom as a screening rule and concluded that the alternative use of chest x-rays was essential. This finding was confirmed by the Kenyan survey [5] which showed a sensitivity of 100% when using chest x-rays combined with symptom screening. Both studies concluded that symptoms alone were insufficient for screening in a prevalence survey, but could be valuable as part of active case finding. Our study did not include chest x-ray data, but demonstrated an increase in sensitivity by using our rule. The purpose of our screen was to see what can be achieved in a household/community setting where only symptom screening can be done by for instance a community health worker to identify adults at relatively high risk of having TB. It is therefore worth investigating using the rule for systematic screening in a high TB/HIV setting, since doing chest x-rays on everyone in the general population is impractical, especially by community healthcare workers. It is also not programmatically cost-effective, as was shown in Botswana when the cost-effectiveness of symptom screening alone was compared to symptom screening in combination with chest x-rays [18].

National TB prevalence surveys have been completed in sub-Saharan Africa (e.g. Zambia and Malawi) and are planned in countries with high TB prevalence (e.g. SA). These surveys screen according to WHO recommendations (symptom screening and chest X-ray) and diagnosis is based on two sputum samples; all surveys use cough ≥2 weeks as part of the screen; some also use other symptoms to determine eligibility for sputum examination. Data from national TB prevalence surveys in which symptoms beyond cough ≥2 weeks were included as part of screening (e.g. Malawi survey) are a resource for comparing the performance of alternative symptom screening rules.

### Limitations

A limitation of using prevalence survey data, especially the ZAMSTAR surveys where cultures were done on sputum samples from every participant without confirmation of clinical disease with chest x-rays, could be transient organism excretion with transient positive cultures [19]. Such participants are usually not ill and although for the purpose of a prevalence estimate, all culture positive samples are indicative of prevalence, they may not be transmitting *M*.*tb*. It is not possible to know for certain what proportion of culture positive participants has transient organism excretion, but based on Zambian data it could be 15–20% [7]. In addition, self-reported data inherently include recall and social desirability biases, which is another limitation of survey methodology, and there could also have been misclassification of some HIV-positive participants as HIV-negative since not every participant had an HIV test result.

### Recommendation

Based on our findings, high TB and HIV burden countries should consider using a symptom screen of three of six TB symptoms or two or more among cough <2 weeks, night sweats and weight loss in addition to cough ≥2 weeks in the context of systematic screening in the general population. In future, national prevalence survey data from other countries could be used to estimate the performance of alternative screening rules.

## Supporting information

S1 File Individual symptoms as predictors, odds ratios and 95% CI restricted to individuals without cough ≥2 weeks in South Africa (training data) (Table A). Counts of symptoms as predictors, odds ratios and 95% CI restricted to individuals without cough ≥2 weeks in South Africa (training data) (Table B).
(DOCX)
