# Supplementary material for: Symptom screening rules to identify active pulmonary tuberculosis: Findings from the Zambian South African Tuberculosis and HIV/AIDS Reduction (ZAMSTAR) trial prevalence surveys
Source: PLoS One. 2017 Mar 3;12(3):e0172881. doi: 10.1371/journal.pone.0172881 (PMC5336223; doi:10.1371/journal.pone.0172881)
Supplement: S1 File — Individual symptoms as predictors, odds ratios and 95% CI restricted to individuals without cough ≥2 weeks in South Africa (training data) (Table A). Counts of symptoms as predictors, odds ratios and 95% CI restricted to individuals without cough ≥2 weeks in South Africa (training data) (Table B). (DOCX) [file pone.0172881.s001.docx]

**Table A.**  **Individual symptoms as predictors, odds ratios and 95% CI restricted to individuals without cough ≥2 weeks in South Africa (training data)**

|  | **% with symptom** | **TB/total** | **%** | **Odds ratio** | **95% CI** |
| --- | --- | --- | --- | --- | --- |
| **All individuals, regardless of HIV status** | | | | | |
| **Total with TB** |  | 276/14154 | 2.0 |  |  |
| **Individual symptoms** |  |  |  |  |  |
| Cough <2 weeks - No |  | 233/13057 | 1.8 |  |  |
| - Yes | 7.8 | 43/1097 | 3.9 | **2.2** | [1.2-4.1] |
| Shortness of breath - No |  | 246/13117 | 1.9 |  |  |
| - Yes | 7.3 | 30/1037 | 2.9 | **1.6** | [1.1-2.2] |
| Fever - No |  | 218/11799 | 1.9 |  |  |
| - Yes | 16.6 | 58/2355 | 2.5 | **1.3** | [0.9-2.0] |
| Night sweats - No |  | 209/12129 | 1.7 |  |  |
| - Yes | 14.3 | 67/2025 | 3.3 | **2.0** | [1.4-2.8] |
| Loss of weight - No |  | 212/12351 | 1.7 |  |  |
| - Yes | 12.7 | 64/1803 | 3.6 | **2.1** | [1.6-2.7] |
| Chest pain - No |  | 248/12944 | 1.9 |  |  |
| - Yes | 8.5 | 28/1210 | 2.3 | **1.2** | [0.8-1.8] |
| **Individuals known to be HIV negative** | | | | | |
| **Total with TB** |  | 104/7164 | 1.5 |  |  |
| **Individual symptoms** |  |  |  |  |  |
| Cough <2 weeks - No |  | 89/6573 | 1.4 |  |  |
| - Yes | 8.2 | 15/591 | 2.5 | **1.9** | [0.9-4.2] |
| Shortness of breath - No |  | 96/6600 | 1.5 |  |  |
| - Yes | 7.9 | 8/564 | 1.4 | **1.0** | [0.7-1.4] |
| Fever - No |  | 88/5894 | 1.5 |  |  |
| - Yes | 17.7 | 16/1270 | 1.3 | **0.8** | [0.6-1.1] |
| Night sweats - No |  | 73/6134 | 1.2 |  |  |
| - Yes | 14.4 | 31/1030 | 3.0 | **2.6** | [1.8-3.7] |
| Loss of weight - No |  | 81/6232 | 1.3 |  |  |
| - Yes | 13.0 | 23/932 | 2.5 | **1.9** | [1.2-3.2] |
| Chest pain - No |  | 94/6502 | 1.5 |  |  |
| - Yes | 9.2 | 10/662 | 1.5 | **1.0** | [0.4-2.5] |

**Table B.**  **Counts of symptoms as predictors, odds ratios and 95% CI restricted to individuals without cough ≥2 weeks in South Africa (training data)**

|  | **% with symptom** | **TB/total** | **%** | **Odds ratio** | **95% CI** |
| --- | --- | --- | --- | --- | --- |
| **All individuals, regardless of HIV status** | | | | | |
| **Count of symptoms** |  |  |  |  |  |
| 0 | 53.6 | 148/9092 | 1.6 |  |  |
| 1 | 18.8 | 52/2508 | 2.1 | 1.3 | [0.9-1.7] |
| 2 | 11.2 | 31/1353 | 2.3 | 1.4 | [1.0-2.1] |
| 3 | 6.5 | 18/689 | 2.6 | 1.6 | [0.9-2.8] |
| 4+ | 9.8 | 27/512 | 5.3 | 3.4 | [2.3-4.8] |
| 1 or more vs none |  | 148/9092 | 1.6 |  |  |
|  | 35.8 | 128/5062 | 2.5 | 1.6 | [1.2-2.0] |
| 2 or more vs <2 |  | 200/11600 | 1.7 |  |  |
|  | 18.0 | 76/2554 | 3.0 | 1.7 | [1.3-2.3] |
| 3 or more vs <3 |  | 231/12953 | 1.8 |  |  |
|  | 8.5 | 45/1201 | 3.8 | 2.1 | [1.6-2.9] |
| 4 or more vs <4 |  | 249/13642 | 1.8 |  |  |
|  | 3.6 | 27/512 | 5.3 | 3.0 | [2.1-4.2] |
| **Count of symptoms, among cough, weight loss, night sweats (CSW)** |  |  |  |  |  |
| 0 | 61.6 | 170/10472 | 1.6 |  |  |
| 1 | 19.6 | 54/2593 | 2.1 | 1.3 | [0.9-1.8] |
| 2 | 13.0 | 36/935 | 3.9 | 2.4 | [1.7-3.5] |
| 3 | 5.8 | 16/154 | 10.4 | 7.0 | [4.4-11.1] |
| 1 or more vs <1 |  | 170/10472 | 1.6 |  |  |
|  | 38.4 | 106/3682 | 2.9 | 1.8 | [1.4-2.3] |
| 2 or more vs <2 |  | 224/13065 | 1.7 |  |  |
|  | 18.8 | 52/1089 | 4.8 | 2.9 | [2.1-3.9] |
| 3 vs <3 |  | 260/14000 | 1.9 |  |  |
|  | 5.8 | 16/154 | 10.4 | 6.1 | [3.9-9] |
| **Count of symptoms, among cough, weight loss, night sweats, fever (CSWF)** |  |  |  |  |  |
| 0 | 67.5 | 154/9547 | 1.6 |  |  |
| 1 | 18.7 | 55/2653 | 2.1 | 1.3 | [0.9-1.8] |
| 2 | 9.5 | 34/1339 | 2.5 | 1.6 | [1.1-2.3] |
| 3 | 3.6 | 23/511 | 4.5 | 2.9 | [1.8-4.5] |
| 4 | 0.7 | 10/104 | 9.6 | 6.5 | [3.8-11.2] |
| 1 or more vs <1 |  | 154/9547 | 1.6 |  |  |
|  | 32.6 | 122/4607 | 2.7 | 1.7 | [1.3-2.1] |
| 2 or more vs <2 |  | 224/13065 | 1.7 |  |  |
|  | 13.8 | 209/12200 | 1.7 | 2.0 | [1.5-2.7] |
| 3 or more vs <3 |  | 243/13539 | 1.8 |  |  |
|  | 4.4 | 33/615 | 5.4 | 3.1 | [2.3-4.3] |
| 4 vs <4 |  | 266/14050 | 1.9 |  |  |
|  | 0.7 | 10/104 | 9.6 | 5.5 | [3.2-9.4] |
| **Individuals known to be HIV negative** | | | | | |
| **Count of symptoms** |  |  |  |  |  |
| 0 | 61.7 | 50/4418 | 1.1 |  |  |
| 1 | 19.5 | 25/1399 | 1.8 | 1.6 | [1.0-2.4] |
| 2 | 10.2 | 15/732 | 2.1 | 1.8 | [1.0-3.2] |
| 3 | 5.1 | 10/364 | 2.8 | 2.5 | [1.2-4.9] |
| 4+ | 3.5 | 4/251 | 3.9 | 1.4 | [0.6-3.6] |
| 1 or more vs none |  | 50/4418 | 1.1 |  |  |
|  | 38.3 | 54/2746 | 2.0 | 1.8 | [1.2-2.5] |
| 2 or more vs <2 |  | 75/5817 | 1.3 |  |  |
|  | 18.8 | 29/1347 | 2.2 | 1.7 | [1.1-2.6] |
| **3 or more vs <3** |  | 90/6549 | 1.4 |  |  |
|  | 8.6 | 14/615 | 2.3 | 1.7 | [1.0-2.9] |
| 4 or more vs <4 |  | 100/6913 | 1.5 |  |  |
|  | 3.5 | 4/251 | 1.6 | 1.1 | [0.4-2.8] |
| **Count of symptoms, among cough, weight loss, night sweats (CSW)** |  |  |  |  |  |
| 0 | 72.7 | 59/5211 | 1.1 |  |  |
| 1 | 19.9 | 25/1424 | 1.8 | 1.6 | [1.0-2.4] |
| 2 | 6.4 | 16/458 | 3.5 | 3.2 | [1.8-5.5] |
| 3 | 1.0 | 4/71 | 5.6 | 5.2 | [2.0-13.9] |
| 1 or more vs <1 |  | 59/5211 | 1.1 |  |  |
|  | 27.3 | 45/1953 | 2.3 | 2.1 | [1.4-3.0] |
| 2 or more vs <2 |  | 84/6635 | 1.3 |  |  |
|  | 7.4 | 20/529 | 3.8 | 3.1 | [1.8-5.1] |
| 3 or more vs <3 |  | 100/7093 | 1.4 |  |  |
|  | 1.0 | 4/71 | 5.6 | 4.2 | [1.6-10.8] |
| **Count of symptoms, among cough, weight loss, night sweats, fever (CSWF)** |  |  |  |  |  |
| 0 | 65.5 | 52/4689 | 1.1 |  |  |
| 1 | 20.4 | 28/1462 | 1.9 | 1.7 | [1.1-2.7] |
| 2 | 10.1 | 17/724 | 2.4 | 2.1 | [1.3-3.7] |
| 3 | 3.4 | 5/243 | 2.1 | 1.9 | [0.8-4.6] |
| 4 | 0.6 | 2/46 | 4.4 | 4.1 | [1.1-15.3] |
| 1 or more vs <1 |  | 52/4689 | 1.1 |  |  |
|  | 34.6 | 52/2475 | 2.1 | 1.9 | [1.3-2.8] |
| 2 or more vs <2 |  | 80/6151 | 1.3 |  |  |
|  | 14.1 | 24/1013 | 2.4 | 1.8 | [1.2-2.9] |
| 3 or more vs <3 |  | 97/6875 | 1.4 |  |  |
|  | 4.0 | 7/289 | 6.7 | 1.7 | [0.9-3.5] |
| 4 vs <4 |  | 102/7118 | 1.4 |  |  |
|  | 0.6 | 2/46 | 1.9 | 3.1 | [0.8-11.9] |
